# A Narrative Review: Actigraphy as an Objective Assessment of Perioperative Sleep and Activity in Pediatric Patients

**DOI:** 10.3390/children4040026

**Published:** 2017-04-18

**Authors:** Nicole Conrad, Joelle Karlik, Amy Lewandowski Holley, Anna C. Wilson, Jeffrey Koh

**Affiliations:** 1 Department of Anesthesiology and Perioperative Medicine, Oregon Health and Science University (OHSU), Portland, OR 97239, USA; karlikj@ohsu.edu (J.K.); kohj@ohsu.edu (J.K.); 2 Department of Pediatrics, Oregon Health and Science University (OHSU), Portland, OR 97329, USA; holleya@ohsu.edu (A.L.H.); longann@ohsu.edu (A.C.W.)

**Keywords:** actigraphy, pediatric, children, sleep, activity, perioperative, postoperative, anesthesia, surgery

## Abstract

Sleep is an important component of pediatric health and is crucial for cognitive development. Actigraphy is a validated, objective tool to capture sleep and movement data that is increasingly being used in the perioperative context. The aim of this review is to present recent pediatric studies that utilized actigraphy in the perioperative period, highlight gaps in the literature, and provide recommendations for future research. A literature search was completed using OVID and PubMed databases and articles were selected for inclusion based on relevance to the topic. The literature search resulted in 13 papers that utilized actigraphic measures. Results of the review demonstrated that actigraphy has been used to identify predictors and risk factors for poor postoperative sleep, examine associations among perioperative pain and sleep patterns, and assess activity and energy expenditure in both inpatient and outpatient settings. We propose expansion of actigraphy research to include assessment of sleep via actigraphy to: predict functional recovery in pediatric populations, to study postoperative sleep in high-risk pediatric patients, to test the efficacy of perioperative interventions, and to assess outcomes in special populations for which self-report data on sleep and activity is difficult to obtain.

## 1. Introduction

Sleep is an important component of pediatric health and is crucial for cognitive development [1]. Disturbances in sleep cycles impair the immune system, alter stress responses [2,3,4], and are associated with behavioral issues and problems with neurobehavioral functioning [5]. Poor sleep in youth also contributes to the development of chronic disease states such as obesity [6] and can impact future quality of life [7].

Research in adults has shown that sleep disturbances are common in the perioperative period. Sleep–wake patterns and circadian rhythms are significantly disturbed by surgery [8,9]. In addition, perioperative sleep disturbances predict poor outcomes including higher postoperative pain [10,11], poorer physical function and emotional well-being [12,13] and impaired functional recovery [14].

While on average over 4 million surgical pediatric procedures are performed annually in the United States [15,16], there is limited pediatric data on the effect of sleep and activity on key postoperative outcome variables such as pain and functional recovery [17]. Emerging evidence shows that sleep disturbances are common in children after surgery and poor postoperative sleep is associated with worse postoperative pain control [18,19].

Self-report measures of sleep are frequently used with children and their parents, but are limited by inaccuracies and bias [20]. Actigraphy uses a watch-like or hip-mounted accelerometer device; this validated and objective tool captures sleep and movement data [21]. Actigraphy can be particularly useful in studying perioperative function in children, as it is an objective measure that can unobtrusively capture data continuously in the home or hospital setting [22]. Pediatric perioperative research has only begun to examine associations among actigraphic measures, patient characteristics, and perioperative variables.

The aims of this narrative review are to: summarize relevant findings in pediatric research utilizing actigraphy in the perioperative period, highlight gaps in the literature, and provide recommendations for future research. This review does not follow the rigorous criteria for a systematic review or meta-analysis. Additionally, the validity and accuracy of actigraphy as a measure within the reviewed studies is not specifically evaluated and is beyond the scope of this review.

A 2013 systematic review by Madsen et al. previously summarized actigraphic research in patients undergoing surgical procedures [23]. While Madsen et al.’s review included a few pediatric studies, it is largely focused on adults. Moreover, the current review includes recent pediatric studies that utilized actigraphy and were not included in Madsen et al.’s review.

## 2. Results

### 2.1. Ambulatory Surgery Impacts Postoperative Sleep and Activity in Youth

Studies using actigraphy to examine sleep in the postoperative setting show that surgery has significant effects on sleep and activity in pediatric patients. Three out of four studies that examined postoperative sleep reported sleep disturbances [18,19,24], ranging from 20–47% of patients. Sleep disturbances included decreased sleep percentage and lower sleep efficiency. Paradoxically, one study comparing postoperative sleep in adolescent surgical patients compared to healthy controls showed that postoperative patients actually had longer sleep hours and better sleep efficiency [25]. In terms of activity measurements, two studies reported significant decreases in motor activity and hyperactivity postoperatively [26,27].

### 2.2. Effect of Perioperative Interventions on Postoperative Sleep

Actigraphy has also been used as a research tool for comparing anesthetic variables and sleep outcomes. Two pediatric studies were identified that looked at different anesthetic techniques. One study compared the intraoperative use of two different inhaled anesthetic agents, sevoflurane and halothane when examining postoperative sleep and behavior measured via actigraphy. There were no significant differences in postoperative behavior changes, pain scores, analgesic requirements or actigraphic variables, such as nighttime awakenings or sleep percentage [28]. A more recent study examined the effect of the use of the preoperative anxiolytic, midazolam, on postoperative sleep and found no significant postoperative sleep changes between the study and control group [29]. Another study looked at two different methods of postoperative pain medication administration (either around the clock medication administration or usual administration pattern which allowed for uninterrupted sleep) after tonsillectomy and adenoidectomy. Actigraphic results showed no differences in sleep duration between the two study groups [30]. 

### 2.3. Energy Expenditure and Physical Activity in Critically Ill Children

One study identified used actigraphy to assess activity levels in relationship to total energy expenditure in critically ill children. This study examined children admitted to the intensive care unit after sepsis or surgery. Analysis showed that actigraphic measurements strongly correlated with energy expenditure, suggesting actigraphy can be used to understand and monitor energy expenditure in critically ill patients [31]. This highlights the utility of actigraphy to measure activity and related energy expenditure in critically ill children.

### 2.4. Activity Monitoring in Children with Congenital Cardiac Disease

Review of relevant articles identified two studies that examined activity using actigraphy in the congenital cardiac population. In this patient population, actigraphy has been used to assess long-term changes in physical activity in pediatric patients [32,33]. One study showed that after congenital heart surgery, exercise capacity was decreased but overall daily activity pattern stayed within normal ranges [32]. In contrast, a separate long-term follow up study in patients who had previously undergone a Fontan procedure (mean time frame of eight years postoperatively) reported that patients had decreased activity overall, independent of exercise capacity [33]. These two studies highlight the use of actigraphy as a measure of activity as an assessment tool in patients with congenital heart disease.

### 2.5. Predictors of Poor Postoperative Sleep

Actigraphic assessment of youth in the postoperative setting has also been used to examine risk factors for poor postoperative sleep. Psychological predictors of poor postoperative sleep include aggressive behavior and parental anxiety/worry [24]. Similarly, pediatric patients with poor sleep during the postoperative period (five days) showed higher anxiety prior to surgery particularly greater anxiety at induction, and had a greater increase in anxiety from the preoperative holding room to induction of anesthesia [18]. In children undergoing tonsillectomy and adenoidectomy, postoperative sleep decrements were predicted by higher child anxiety at induction and less sociability scores [19].

### 2.6. Risk Factors for Poor Postoperative Pain Control

Actigraphy data in youth also shows strong associations among sleep and pain. Studies demonstrate that poorer postoperative sleep, such as decreased sleep efficiency, is associated with worse postoperative pain control [18,19,24]. Rabbitts et al. showed that parental pain catastrophizing and shorter preoperative sleep duration predicted worse postoperative pain [17].

## 3. Discussion

### 3.1. Agenda for Future Research

The studies reviewed collectively highlight potential applications for using actigraphy in the perioperative setting. Specifically, actigraphy has been used to identify predictors and risk factors for poor postoperative sleep, examine associations among perioperative pain and sleep patterns and assess activity and energy expenditure in both inpatient and outpatient settings [17,19,32,33]. Below we outline several areas identified throughout this review that might be valuable to address in future research.

#### 3.1.1. Expand Use of Actigraphy to Assess Functional Recovery in Pediatric Patients

In adults, sleep has not only been associated with worse pain control, but also with delayed functional recovery [14]. Research has yet to explore how sleep and activity assessed via actigraphy might be used to predict functional recovery in pediatric populations. Do pediatric patients with sleep disturbances take longer to heal or return to normal activity patterns? Future research examining functional recovery in pediatric patients should focus on prospective, longitudinal studies examining specific measures of postoperative healing such as return to normal activities, general well being, fatigue, and health-related quality of life. Actigraphy could be used to assess how different anesthetic or surgical interventions (such as laparoscopic versus open surgery or varying anesthetic agents) affect functional recovery.

#### 3.1.2. Improve Perioperative Outcomes with Preoperative Sleep Interventions

Multiple studies identified predictors of poor postoperative sleep that include both patient and parental characteristics (e.g., sleep hygiene, parental anxiety). More research is needed to use predictors of poor postoperative sleep to identify high-risk pediatric patients. Novel preoperative interventions could target these patients preoperatively and actigraphy can be used to assess the efficacy of interventions during the immediate and extended perioperative period. This could be important for improving recovery and postoperative pain as the reviewed articles demonstrate the importance of both pre and postoperative sleep on postoperative pain control.

#### 3.1.3. Optimize Management of Patients in the Pediatric Inpatient Setting

Actigraphy could be used more widely in the inpatient setting to assess and improve inpatient sleep–wake cycles. Data obtained from actigraphy could inform dosing of analgesic medications and sedation infusions to minimize over-sedation and adverse side effects such as alterations in day–night cycles and immobility. Actigraphy, when combined with other monitoring methods, could also be studied to attempt to differentiate between pain and delirium in the intensive care setting. Actigraphy could be used to identify patients at increased risk for deep vein thrombosis by measuring decreased activity or prolonged periods of inactivity.

#### 3.1.4. Perioperative Assessment Tool for Special Populations

Another use for actigraphy in the perioperative period is to assess outcomes in special population groups where self-report and objective data is difficult to obtain such as in patients with neurodevelopmental disorders. For example, actigraphy has already been used to assess sleep in children with autism [34] in the perioperative setting.

#### 3.1.5. Improve Perioperative Care with Accessible Technologies

While consumer devices that measure movement and sleep (e.g., Fitbit) are widely available, there is little data on reliability for sleep monitoring in pediatric patients when compared to actigraphy [35]. A recent study in pediatric patients showed similar sleep measurements when comparing an actiwatch versus a commercial wrist-accelerometer [36]. The next step is to utilize these consumer devices in the clinical research setting. Eventually, data from these more accessible devices collected perioperatively might help inform patient care.

## 4. Materials and Methods 

A literature search was completed with the assistance of a research librarian, using OVID and PubMed databases, with the following search terms: Actigraphy (actigraphic; accelerometer), AND children (children; adolescents), AND surgery (surgery; perioperative; pre/postoperative, anesthesia). Abstracts and articles were screened by two of the manuscript’s authors (N.C. and A.C.W.) and selected for inclusion based on relevance to the topic. Thirteen studies were selected for inclusion in the review. There were no disagreements among authors regarding inclusion. Subsequently, six research themes within the studies were identified and agreed upon by the two authors. Studies were grouped by the following topics by similar topics in the summary below.

## 5. Conclusions

Research using actigraphy has the potential for optimizing pediatric perioperative care and could be an important addition to the perioperative surgical home model where standardization and improved individual care is the goal [34]. More investigation is needed to determine if actigraphy could be used as a research tool to help identify intervention targets for improved patient outcomes such as decreased admission lengths, reduced rates of surgical complications, and overall improved patient safety. The application of actigraphic technologies in research and clinical care settings may ultimately allow the anesthesiologist to tailor anesthetic care to optimize the patient’s perioperative experience.
